# Changes in the Circadian Rhythm in Patients with Primary Glaucoma

**DOI:** 10.1371/journal.pone.0062841

**Published:** 2013-04-29

**Authors:** Huaizhou Wang, Ye Zhang, Jianming Ding, Ningli Wang

**Affiliations:** 1 Beijing Tongren Eye Center, Beijing Tongren Hospital, Capital Medical University, Beijing Ophthalmology and Vision Science Key Lab, Beijing, China; 2 Department of Physiology, Brody School of Medicine, East Carolina University, Greenville, North Carolina, United States of America; National Institute of Environmental and Health Sciences, United States of America

## Abstract

**Purpose:**

The current study was undertaken to investigate whether glaucoma affects the sleep quality and whether there is any difference between patients with primary glaucoma (primary open angle glaucoma, POAG and primary angle-closure glaucoma, PACG) and healthy subjects, using a validated self-rated questionnaire, the Pittsburgh Sleep Quality Index (PSQI).

**Methods:**

The sleep quality of patients with POAG and PACG was tested against normal controls. Subjects were divided into three sub-groups according to age. Differences in the frequency of sleep disturbances (PSQI score >7) were assessed. The differences of sleep quality within the three groups and within the POAG group depending on the patients’ intraocular pressure (IOP) and impairment of visual field (VF) were also studied.

**Results:**

92 POAG patients, 48 PACG patients and 199 controls were included. Sleep quality declined with age in control and POAG group (tendency chi-square, P<0.05). The prevalence of sleep disturbances was higher in POAG and PACG group than in the control group, the differences were statistically significant. The prevalence of sleep disturbances was higher in patients with PACG, compared to POAG patients in the age interval of 61–80. In POAG group, the ratio of patients with sleep disorders increased with augmented impairment of VF, but the differences were not statistically significant (χ^2^-test, P>0.05). No significant differences were found in POAG group between patients with a highest IOP in daytime and at nighttime (χ^2^-test, P>0.05).

**Conclusions:**

The prevalence of sleep disorders was higher in patients with POAG and PACG than in controls. PACG patients seemed to have a more serious problem of sleep disorders than POAG patients between 61 to 80 years old. No correlation was found between the prevalence of sleep disorders and impairment of VF or the time when POAG patients showed a highest IOP.

## Introduction

Human eyes mediate both image-forming and non-image-forming visual functions. Classic photoreceptors including rods and cones, superior collicular retinal ganglion cells (scRGCs) and the neurons in the visual cortex are responsible for the image-forming visual pathway that produce vision. It is now well established that intrinsically photosensitive retinal ganglion cells (ipRGCs), a specialized subset of retinal ganglion cells, which express the photopigment melanopsin, form the retinohypothalamic tract that directly innervate the suprachiasmatic nucleus of the hypothalamus (SCN) and mediate non-image forming functions of the eye, including the photoentrainment of circadian rhythms to the light-dark cycle, the regulation of pupillary light reflex, negative masking and regulation of melatonin secretion [1]–[18].

Glaucoma is the second leading cause of blindness, after cataract, and the first cause of irreversible blindness worldwide [19]. This ocular degenerative disease affects the RGCs and eventually causes optic nerve atrophy, visual impairment and blindness by way of axonal loss [20].

Recently, the involvement of ipRGCs as well as regular RGCs in glaucoma has been postulated. Indeed, several studies in animal models of glaucoma have demonstrated a degenerative loss of ipRGCs or melanopsin photopigment leading to impairment of circadian rhythms regulation, pupillary light responses and other non-image-forming functions [1], [11], [21]–[23]. Also, preliminary data in humans suggested that abnormal circadian rhythm of melatonin secretion, light-induced melatonin suppression and reduced pupillary light reflex occur in glaucoma patients [24]–[26]. Furthermore, a high prevalence of sleep disorders, such as insomnia, daytime sleep, sleep apnea can be found in patients with glaucoma [27]–[31].

Therefore, not only the image-forming visual system, but also the non-image-forming visual system is associated with glaucoma. The damage to the latter may have influence on the regulation of circadian rhythm and other non-image-forming functions. In humans, the most characteristic activity of circadian rhythm is the sleep/wake cycle. Hence, our hypothesis is that in patients with glaucoma, different degrees of sleep disturbances may occur as a result of the characteristic damage to ipRGCs. So far, previous researchers have supported this hypothesis on some level, but more evidence was warranted. Furthermore, it is still not clear whether there is any difference in the prevalence of sleep disorders within glaucoma patients with different degrees of glaucomatous impairments. Thus, we investigated and compared different degrees of sleep disturbances among patients with primary open angle glaucoma (POAG), patients with primary angle-closure glaucoma (PACG) and normal people without severe ocular or systemic diseases, using the Pittsburgh Sleep Quality Index (PSQI), in order to find out changes of sleep quality of glaucoma patients and possible related factors. The PSQI is a validated self-rated questionnaire that assesses sleep quality and disturbances during a period of one month and has been used in many clinical conditions and researches [32]–[35]. In PSQI, 19 individual items generate 7 component scores: subjective sleep quality, sleep latency (time needed to fall asleep), sleep duration, habitual sleep efficiency (proportion between total sleep time and time in bed), sleep disturbances (i.e. waking up during night), use of sleeping medication, and daytime dysfunction (difficulty to stay awake during the day). The sum of the scores of the 7 items yields a global score with a possible range of 0–21 [32]. A global PSQI score higher than 7 yields a diagnostic sensitivity of 98.3% and specificity of 90.2% (Kappa = 0.89, P<0.01) in distinguishing good and poor sleepers in Chinese population [34], hence we used a cutoff score of 7 to consider the existence of sleep disturbances in our current study.

## Methods

### Subjects

In this study, 99 patients with POAG and 55 patients with PACG were recruited consecutively in the Department of Ophthalmology at Beijing Tongren Hospital, Beijing, China, over seven months. All the patients were diagnosed by more than two ophthalmologists in accordance with the diagnostic criteria for POAG and PACG. A control group of 210 healthy subjects without any severe systemic or ocular disease were recruited from Desheng community of Beijing, China, over three months. All subjects understood the purpose of the study and volunteered to participate. The subjects were excluded if they have had any kind of intraocular surgery or laser treatment within three months prior to this study or if they have a personal history of any other ocular diseases such as retinitis pigmentosa (RP), systemic diseases including psychiatric illness, endocrine illness, drug or alcohol abuse, night work or transmeridian travel in the previous three months, that might affect the results. Patients with POAG or PACG were required to have a stable condition including an IOP lower than 40mmHg in both eyes, no self-discomfort such as ophthalmalgia, headache, or nausea.

Subjects of the three groups were divided according to their age into any of the three age interval sub-groups, the first being 18 to 40-year-old and the last being 61 to 80-year-old.

### Evaluation of the Subjective Sleep Quality

Sleep quality of each subject in the three groups (POAG group, PACG group and control group) was assessed by means of the PSQI questionnaire. Sleep was considered to be disturbed if the global PSQI score was higher than 7. Frequency of sleep disturbances in each group was assessed. The differences between either two of the three groups were compared according to the age-related sub-groups.

**Figure 1:**
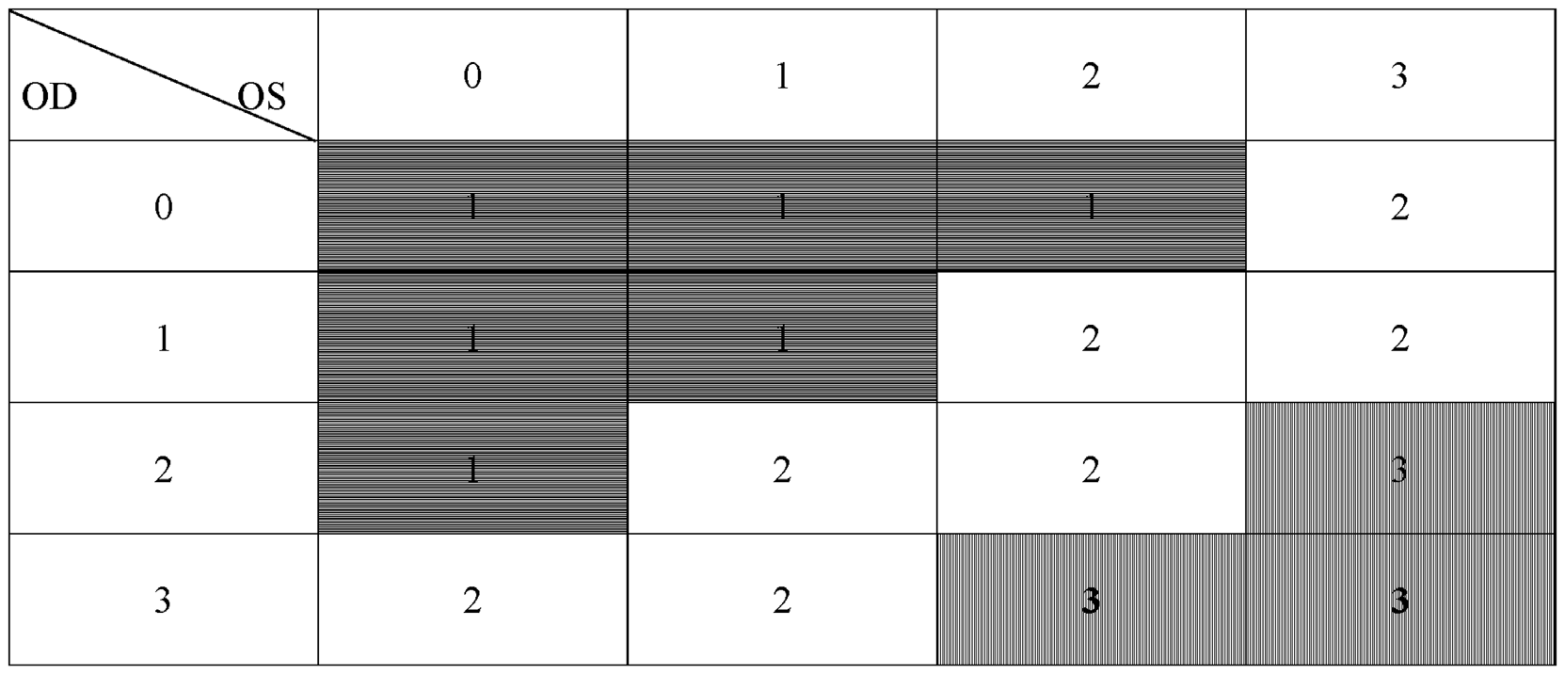
The classification of visual field in POAG patients. *Level 1 = Horizontal stripes, level2 = blank and level 3 = Vertical stripes.

Meanwhile, in the POAG group, patients were classified into three further sub-groups as mild, moderate, and severe visual field loss, according to their different degrees of visual field loss. Monocular visual field loss can be graded into four levels, including no visual field impairment (defined as 0), early-stage defects such as paracentral scotomas, nasal step defects et al (defined as 1), advanced-stage defects like arcuate scotoma (defined as 2) and end-stage defects including a small central island and a temporal island (defined as 3) [36]–[37]. The classification of binocular visual field defects is as following: if the visual field impairment of one eye is classified to be 0 and the other eye to be 0, 1 or 2, or both eyes 1, then the binocular visual field defects is graded to be level 1. If the visual field impairment of either eye is classified to be 2 and the other eye to be 2 or 3, or both eyes 3, then the binocular visual field defects is graded to be level 3. And the rest of the conditions are graded to be level 2. All of this information is shown in Figure 1. Differences between these three sub-groups were assessed to decide the relationship between sleep disturbances and impairment of visual field. Moreover, we evaluated the prevalence of sleep disturbances in patients who showed a highest intraocular pressure (IOP) in daytime defined as 6∶00AM to 10∶00PM within the same day and at nighttime defined as 10∶00PM to 6∶00AM in the next day in the age groups of 41–60 and 61–80 years old respectively, by means of 24-hour IOP measurement. This was done to compare the frequency of sleep disturbances between the two sub-groups within the same age interval to investigate if IOP is an important factor that affects sleep quality in POAG patients. It has been proved in previous studies that IOP was positively related with central corneal thickness (CCT) [38]–[40]. Therefore, the actual IOP level of each subject in our research was obtained by correction of IOP according to CCT, as can be seen in Table 1 [41]. The reason why POAG patients with an age less than 41 were not included for IOP analysis is that only 10 patients younger than 41 received 24-hour IOP measurement.

**Table 1:** Correction values for IOPs based on CCT.

CCT in microns	IOP correction in mm Hg
445	+7
455	+6
465	+6
475	+5
485	+4
495	+4
505	+3
515	+2
525	+1
535	+1
545	0
555	−1
565	−1
575	−2
585	−3
595	−4
605	−4
615	−5
625	−6
635	−6
645	−7

### Statistics

Statistical analysis was performed using SPSS 17.0 for Windows. Continuous variables were expressed as mean values ± standard deviation (M ± SD) and analyzed by using the independent samples t-tests, one-way ANOVA and nonparametric test, while categorical variables were presented as frequency and percentage and were assessed by means of the χ^2^-test and tendency Chi-square. The level of significance was 0.05 in all statistical tests.

## Results

**Figure 2:**
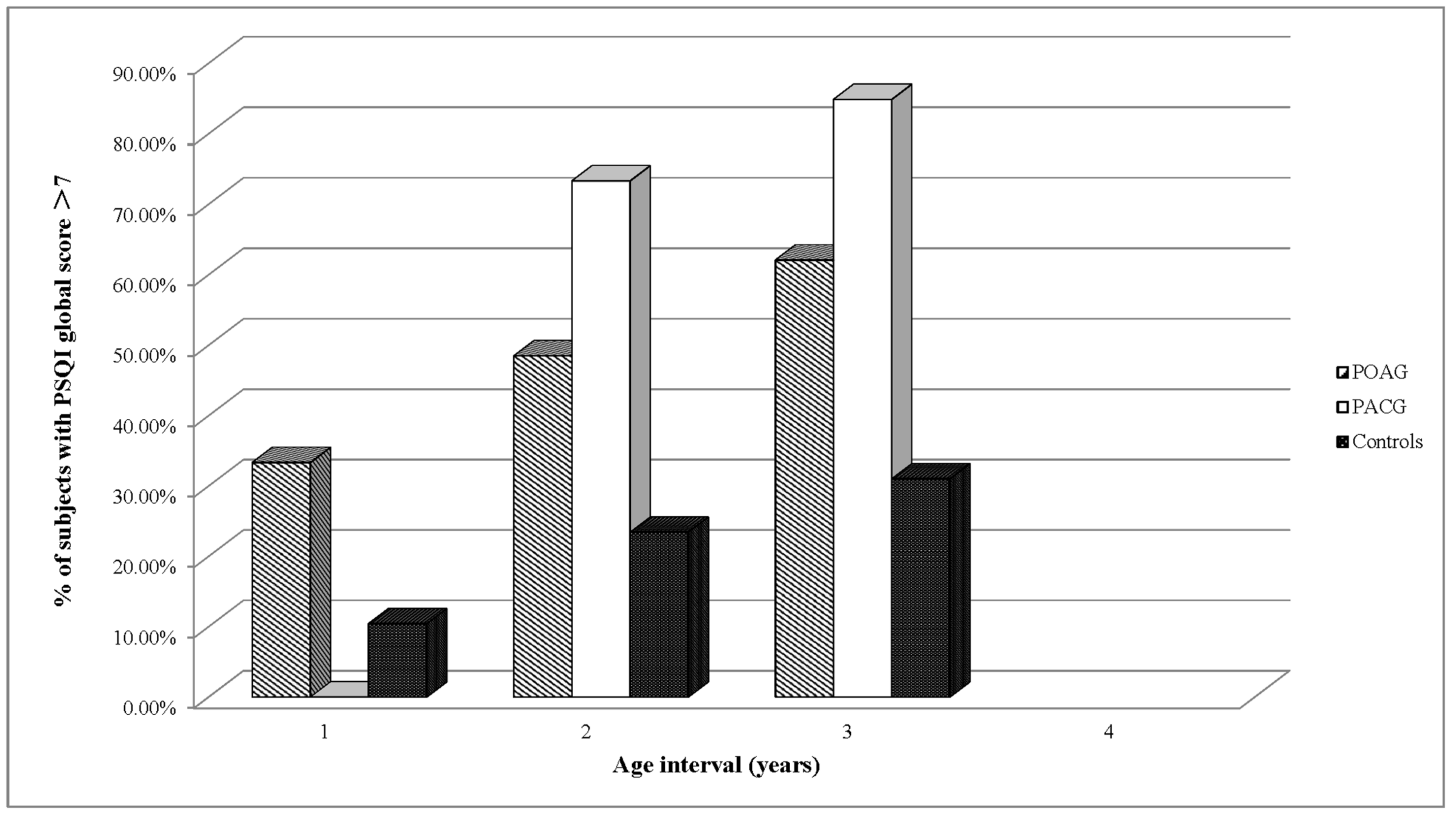
Prevalence of sleep disorders (percentage of subjects with PSQI score>7) among people with POAG, PACG and healthy controls at different age intervals.

Eventually, 92 POAG patients, 48 PACG patients and 199 controls with a complete and valid questionnaire were included. The demographic data of subjects in three groups are shown in Table 2. Figure 2 shows prevalence of sleep disorders (PSQI score >7) among subjects affected by POAG and PACG and normal controls. Table 3 indicates the PSQI total score and percentage of subjects with sleep disturbances in three sub-groups of different age intervals of POAG, PACG and controls respectively.

**Table 2:** Demographic data of subjects in POAG, PACG and control group.

Group	Total number	Number of men	Number of women	Age (Median (IQR)) (years old)
POAG	92	57	35	58 (41–66)
PACG	48	20	28	65 (60–70)
Control	199	100	99	52 (37–63)

* IQR means interquartile range.

**Table 3:** PSQI score and percentage of subjects with PSQI score>7 in six sub-groups among POAG patients, PACG patients and controls.

Age group (years old)	POAG			PACG			Controls			
	TN (M/F)	PSQI (M±SD)	NPSD (%)	TN (M/F)	PSQI (M±SD)	NPSD (%)	TN (M/F)	PSQI (M±SD)	NPSD (%)	
18–40	30(20/10)	7.00±2.639	10(33.33%)	3(1/2)	10.33±2.309	2(75%)	48(26/22)	4.83±2.127	5(10.42%)	
41–60	33(21/12)	8.45±3.392	16(48.48%)	15(5/10)	10.20±3.299	11(73.33%)	64(35/29)	5.67±2.637	15(23.44%)	
61–80	29(16/13)	8.13±2.959	18(62.07%)	33(15/18)	10.45±3.280	28(84.85%)	87(39/48)	6.37±3.644	27(31.03%)	

* TN: total number, M/F: male/female, NPSD: number of patients with sleep disorders.

The onset of PACG in patients is often over 40-years-old and rarely below the age of 30 [19], [42]. In this study, there were only three PACG patients below 40-years-old. Therefore the comparison of PACG patients with POAG patients and controls was performed over 40-years-old.

According to the analysis, global sleep quality decreased with age in healthy controls and POAG patients (tendency Chi-square, P<0.05), as shown in Table 4. And there was no significant differences in PSQI total score according to gender for controls at any of the age intervals studied (t-test, P>0.05 in all sub-groups) as shown in Table 5. As can be seen in Table 6, the prevalence of sleep disturbances was higher in patients with POAG than in controls, the differences being statistically significant in each sub-group (χ^2^-test, P<0.05). And the prevalence of sleep disturbances was higher in patients with PACG than in controls among sub-groups of 41–60 and 61–80 age intervals, the differences being significant (χ^2^-test, P<0.001). Moreover, the prevalence of sleep disturbances was higher in patients with PACG, compared to POAG patients in the age interval of 61–80 (χ^2^-test, P<0.05), but not in the age interval of 41–60 (χ^2^-test, P>0.05).

**Table 4:** The impact of age on the sleep quality in POAG patients PACG patients and Controls (Tendency Chi-square).

Agegroup(yearsold)	Controls			POAG				PACG				
	NPSD/NPWSD	PPSD	P value	NPSD/NPWSD		PPSD	P value	NPSD/NPWSD	PPSD		P value	
18–40	5/43	10.42%	0.0076	10/20	33.33%		0.0271	Not been analyzed				
41–60	15/49	23.44%		16/17	48.48%			11/4		73.33%	0.3434	
61–80	27/60	31.03%		18/11	62.07%			28/5		84.85%		
Total	47/152	23.62%		44/48	47.83%			39/9		81.25%		

* NPSD: number of patients with sleep disorders, NPWSD: number of patients without sleep disorders, PPSD: percentage of patients with sleep disorders.

**Table 5:** T-test for PSQI scores between men and women of different age sub-groups in controls.

Age group (years old)	Number of subjects (male)	Number of subjects (female)	PSQI (M±SD) (male)	PSQI (M±SD) (female)	P value
18–40	26	22	5.70±2.73	5.63±2.31	0.905
41–60	35	29	6.62±3.55	7.67±3.19	0.108
61–80	39	48	7.06±3.43	8.24±4.19	0.063

**Table 6:** χ^2^-test for comparison of prevalence of sleep disorder between POAG patients & controls and PACG patients & controls.

Age group (years old)	POAG & Control	PACG & Control	POAG & PACG
	P value	P value	P value
18–40	0.013	Not been analyzed	Not been analyzed
41–60	0.012	<0.001	0.1077
61–80	0.003	<0.001	0.0408

* PPSD: percentage of patients with sleep disorders.

In the POAG group, the ratio of people with sleep disorders grew with the augmentation of the visual field impairment, with the differences not being statistically significant (χ^2^-test, P>0.05), as can be seen in Table 7. Also, no significant differences were found between patients with a highest IOP in daytime and patients with a highest IOP at nighttime in POAG group of both age intervals (χ^2^-test, P>0.05), as presented in Table 8.

**Table 7:** χ^2^-test for POAG patients with different levels of visual field loss.

Sub-groups (levels of VF loss)	NPSD	NPWSD	TN	PPSD	P value
1	9	16	25	36.00%	0.2361
2	20	22	42	47.62%	
3	15	10	25	60.00%	
Total number	44	48	92	47.83%	

* NPSD: number of patients with sleep disorders, NPWSD: number of patients without sleep disorders, TN: total number, PPSD: percentage of patients with sleep disorders.

**Table 8:** χ^2^-test for POAG patients who showed a highest IOP in day time and night time.

Age group(years old)	Sub-group	IOP range(mmHg)	TN	Men/women	PSQI score(M±SD)	NPSD	PPSD	P value
41–60	Highest IOP in daytime	20.00–32.70	11	7/5	8.18±2.562	5	45.45%	0.500
	Highest IOP innighttime	17.70–31.00	11	6/5	7.45±2.806	4	36.36%	
61–80	Highest IOP in daytime	18.30–31.30	12	8/4	8.58±4.209	7	58.33%	0.623
	Highest IOP innighttime	13.00–27.70	9	5/4	7.00±2.345	5	55.56%	

* TN: total number, NPSD: number of patients with sleep disorders, PPSD: percentage of patients with sleep disorders.

## Discussion

There are many sleep assessment methods that include polysomnography, actiwatch, questionnaire and so on [43]–[44]. We chose the PSQI, which includes advantages over other methods such as the ability to: (a) determine patterns of sleep dysfunction over a 1-month period through assessment of both qualitative and quantitative data; and (b) calculate a simple global score that conveys both the number and severity of sleep problems [32]. The PSQI has been proved to have adequate reliability, good validity and high test-retest reliability, and may be used as a standardized tool for the assessment of subjective sleep quality among individuals of various ages and has wide clinical and research applications [33]–[35], [45]–[46].

The PSQI has been used to assess sleep quality of blind subjects and patients with ocular diseases [47]–[49]. Yang and Li demonstrated that the prevalence of sleep disturbances in patients with glaucoma was higher when compared to healthy controls [30]. However, different types of glaucoma were not analyzed separately and no correlation between IOP or impairment levels of visual field and prevalence of sleep disturbances was studied.

In our research, we found that global sleep quality decreased with age in both healthy controls and POAG patients. With increasing age, the density of the lens increases thereby reducing light transmission, particularly for the short wavelength (blue) light to which the circadian system has been shown to be most sensitive [50]. On the other hand, the occurrence of circadian timing disturbances with age may also be due to neurodegenerative changes in the SCN which may cause decreased regulation function of the non-image-forming system [51].

The prevalence of sleep disorders of POAG and PACG patients was higher than that of controls within the same age intervals, the differences being statistically significant. The severe problem of sleep disturbances of glaucoma patients could be explained by the reduction of the photic input to the circadian system as a result of damage of ipRGCs in glaucoma, as the hypothesis that we proposed. We also found an interesting result that PACG patients have higher prevalence of sleep disturbances comparing to POAG patients in the age interval of 61–80. Why is that? There are some differences in the pathology of PACG and POAG. In some of the PACG patients, acute IOP elevation (up to 60 mmHg or higher) can cause ischemic-reperfusion damage to the whole retina, including not only the ganglion cells, but also other neurons like rods and cons [52]–[54]. In POAG, chronic IOP elevation can cause the ganglion cells apoptosis and axon loss; it has less effect on other neurons in retina than in PACG. So in PACG, the residual ipRGCs may get less input from the residual rods and cons. Also, the mechanisms of RGCs damage in POAG and PACG are different. This may partially explain the fact mentioned above.

Visual field loss is a manifestation caused by damages of RGCs in glaucoma. Characteristic visual field impairments include early-stage defects such as paracentral scotomas, nasal step defects, enlargement of defects like arcuate scotoma and end-stage defects including a small central island and a temporal island [55]. In this study, we found that the ratio of POAG patients with sleep disorders grew with the aggravation of the impairment of visual field, but the differences were not statistically significant. In other words, no correlation was found between the prevalence of sleep disorders and impairment of visual field in patients with POAG. It has been suggested that more than 40% of nerve fiber loss happens before any visual field defect or a change in the optic disc could be detected in patients with glaucoma [55]. This means that ipRGCs, as well as other RGCs, might have been damaged before any visual field loss occurs in patients with glaucoma. Thus, in this study, different levels of visual field impairment did not represent significant differences of severity of ipRGCs damage that could cause different prevalence of sleep disturbances in patients with POAG. In addition, the negative result may also be attributed to inadequate number of subjects. Further research should include a larger cohort to reach a conclusive finding of the correlation between visual field loss and prevalence of sleep disorder in patients with glaucoma.

Recently, some studies demonstrated that another non-image-forming visual function is impaired in patients with glaucoma as a result of ipRGCs loss. Kankipati and colleagues have found that there is a significant decrease in the ipRGCs-mediated post-illumination pupil response (PIPR) in glaucoma patients when compared to age-matched controls [26]. As the severity of the glaucomatous neuropathy increases, there is a correlated decrease in the PIPR. Feigl and colleagues also demonstrated that this phenomenon could be seen in both patients with moderate and severe glaucoma [56]. These findings together with our results indicate that non-image-forming visual functions are injured in clinical glaucoma patients.

In previous studies, sleep disorders of patients with glaucoma have been attributed to psychological factors or discomforts caused by an elevated IOP during the night [31]. However, statistical analysis of our data shows that there is no correlation between the prevalence of sleep disturbances and the time when patients with POAG exhibit the highest IOP. This might mean that in a certain range of IOP, sleep quality may not get affected by elevated IOP during the night.

Our results clearly show that the sleep quality of patients suffering from POAG and PACG is significantly decreased. The reason for this reduction in sleep quality could be found in the degeneration of ipRGCs mediating the photic input to the SCN in this disease which may cause damages in non-image-forming visual pathway and changes of circadian rhythm. Recognition of this problem provides awareness of the fact that these sets of patients with glaucoma not only have impairment of visual functions, but also suffer from sleep disturbances. Therefore, it may be helpful to improve their sleep quality by treatment with chronotherapeutic approaches (appropriately-timed exposure to bright light which may reset the timing of sleep and wake to the desired times and improve sleep quality), melatonin treatment which can positively influence comorbid circadian misalignment and sleep disorders [57]–[58].

Our study has some limitations that deserve comment. First, sleep problems were assessed by a self-reporting questionnaire and were not confirmed by polysomnographic measurements. However, the PSQI has been largely validated and widely used as a sufficiently precise tool to discriminate between people with or without sleep troubles. In future, a more objective index may be warranted to test the sleep disturbances of glaucoma patients. The second limitation is that we did not perform the lens grading and correct the lens density. In our study, 2 patients (4 eyes) in POAG group, 1 patient (1 eye) in PACG group and 2 controls (4 eyes) have had IOL implantation. Others have normal lens or slight cataract, with a visual acuity of 20/60 or better. Another study showed that, “No significant differences in sleep quality were found among RP-patients or controls depending either on their gender or on the presence of cataracts” [48]. So we neglected the effect of lens on sleep quality in our study. The results will be more precious with the lens grading. The third limitation concerns the question of the real relationship between IOP and sleep disorders. In our study, the highest IOP after correction was below 33mmHg. Hence we couldn’t decide if IOP higher than this may affect the sleep quality of POAG patients or not. As a result, more attention should be paid to the conclusion we made, that within a certain range of IOP, the sleep quality of glaucoma patients may not be affected by an elevated IOP during night.

### Conclusion

Sleep quality declined with age in the control group and the POAG group. The prevalence of sleep disturbances is higher in patients with POAG and PACG than healthy controls; the prevalence of sleep disturbances was higher in patients with PACG, compared to POAG patients in the age interval of 61–80. No correlation was found between the prevalence of sleep disorders and impairment of visual field in POAG patients or the time when POAG patients showed a highest IOP.
